# Do Economic Recessions During Early and Mid-Adulthood Influence Cognitive Function in Older Age?

**DOI:** 10.1136/jech-2013-202843

**Published:** 2013-11-20

**Authors:** Anja K. Leist, Philipp Hessel, Mauricio Avendano

**Affiliations:** 1 University of Luxembourg, Faculty of Language and Literature, Humanities, Arts and Education, Walferdange, Luxembourg; 2 London School of Economics and Political Science, Department of Social Policy, LSE Health and Social Care, London, United Kingdom; 3 Harvard School of Public Health, Department of Social and Behavioral Sciences, Boston, USA

**Keywords:** Cognition, life course epidemiology, employment, elderly

## Abstract

**Background:**

Fluctuations in the national economy shape labour market opportunities and outcomes, which in turn may influence the accumulation of cognitive reserve. This study examines whether economic recessions experienced in early and mid-adulthood are associated with later-life cognitive function.

**Method:**

Data came from 12,020 respondents in 11 countries participating in the Survey of Health, Ageing and Retirement in Europe (SHARE). Cognitive assessments in 2004/5 and 2006/7 were linked to complete work histories retrospectively collected in 2008/9, and to historical annual data on fluctuations in Gross Domestic Product (GDP) per capita for each country. Controlling for confounders, we assessed whether recessions experienced at ages 25-34, 35-44 and 45-49 were associated with cognitive function at ages 50-74.

**Results:**

Among men, each additional recession at ages 45-49 was associated with worse cognitive function at ages 50-74 (b = -0.06, Confidence Interval [CI] -0.11, -0.01). Among women, each additional recession at ages 25-44 was associated with worse cognitive function at ages 50-74 (b_25-34_ = -0.03, CI -0.04, -0.01; b_35-44_= -0.02, CI -0.04, -0.00). Among men, recessions at ages 45-49 influenced risk of being laid-off, whereas among women, recessions at ages 25-44 led to working part-time and higher likelihood of downward occupational mobility, which were all predictors of worse later-life cognitive function.

**Conclusions:**

Recessions at ages 45-49 among men and 25-44 among women are associated with later-life cognitive function, possibly via more unfavourable labour market trajectories. If replicated in future studies, findings may indicate that policies that ameliorate the impact of recessions on labour market outcomes may promote later-life cognitive function.

## Introduction

Mid-life labour market outcomes and working conditions have been shown to predict cognitive function and decline at older age. Occupational class,[1-4] longer working hours,[5] occupational solvent exposures, career trajectories,[3, 6, 7] and occupational complexity at work,[8-10] are all strong predictors of later-life cognitive function. Based on the cognitive reserve framework,[11] these studies hypothesize that working conditions influence the potential to build up cognitive reserve, which in turn influences cognitive performance at later ages. These studies are, however, prone to selection bias, because higher cognitive function may select individuals into more favourable occupations and working environments. For example, previous research suggests that subtle differences in cognitive function early in life may lead to divergent career trajectories.[12] A potential alternative to address this bias is to examine how macro-economic “shocks”, which can be viewed as largely exogenous to cognitive function of the working population, relate to later-life cognitive outcomes. The rationale for this approach is that cohorts affected by negative, unanticipated macro-economic shocks at vulnerable points in their life-course may have less potential to build up cognitive reserve. These effects, however, would be unrelated to their early life-characteristics or other individual factors that affect their individual labour market and cognitive function trajectories.

This hypothesis is supported by evidence that economic hardship during early childhood is associated with lower cognitive function in older age, in particular if additional disadvantages come into play. [13] Similarly, recent evidence suggests that being born during a recession is significantly associated with lower later-life cognitive function. [14] No studies have yet explored whether recessions experienced during working ages may have cumulative effects on cognitive function at older ages. To our knowledge, this is the first study to adopt a life course perspective to assess whether macroeconomic fluctuations experienced during working ages are associated with later cognitive function, and to investigate some of the potential labour market mechanisms that may explain this association.

Macro-economic conditions during working ages may influence accumulation of cognitive reserve via unfavourable working conditions and opportunities in terms of social mobility. In support of this view, evidence indicates that economic recessions experienced in the year of transition from school to work are associated with less favourable career trajectories, higher job instability, reduced earnings and less favourable working conditions in mid-life. [15, 16] [17] So far, no studies have examined whether these working conditions affected by labour market fluctuations around these vulnerable ages may also have long-lasting and permanent negative associations with cognitive function at older age.

In this paper, we examine whether economic recessions experienced in early and mid-adulthood are associated with later cognitive function. We assessed exposure to recessions during early (ages 25-34), mid-age (35-44) and late middle adulthood (45-49) covering the years prior to retirement and related this to cognitive assessments at ages 50-74 in a sample of Europeans in 11 countries. Based on the life-course accumulation of advantages and disadvantages framework,[18] we hypothesized that each additional recession experienced during working ages is associated with worse later-life cognitive function. In addition, we expected recessions experienced during early adulthood to have a stronger association with cognitive function than recessions experienced later in life. To shed light on the potential mechanisms, we also linked data on economic recessions to individual-level data on full employment histories covering occupational and labour market conditions throughout adulthood. To our knowledge, our study provides the first assessment of how macroeconomic fluctuations during adult life relates to cognitive function in older age.

## Methods

### Data

The Survey of Health, Ageing and Retirement in Europe SHARE is a nationally representative survey which has been designed to provide cross-sectional as well as longitudinal information on the health, employment and social conditions of Europeans aged 50+. Specific details on the survey are available elsewhere. [19-22] [23] A German internal review board (IRB) has approved of ethical standards, study design and data collection.[19]

This study is based on data from three waves of SHARE, the last of which contains detailed individual work histories retrospectively collected using the life-grid method.[24] The total sample included 19,473 participants who had enrolled in the study in either 2004/5 or 2006/7 and completed the life-history interview in 2008/9 from 11 countries (Sweden, Denmark, Austria, France, Germany, Switzerland, Belgium, the Netherlands, Spain, Italy, Greece) and had worked at least once during their working life. Respondents from Czech Republic and Poland were not included due to lack of comparable data on GDP before 1990. We further restricted the sample to individuals aged 50 to 74 at the time of first interview to prevent selective attrition due to higher prevalence of cognitive impairment at higher ages (*n* = 14,765). We excluded individuals with missing information on two or more of the cognitive measures (n = 1,345), childhood health, education (*n* = 292), or sampling weights (*n* = 37) and those respondents who never worked (*n* = 1,071). The final sample included a total of 12,020 men and women in 11 countries (Appendix Table 1).

### Cognitive function

Cognitive function was assessed once at the first time individuals were interviewed based on the indicators *verbal fluency* (naming as many animals as possible in one minute), *immediate recall* (immediately recalling as many words as possible from a ten-word list that had been read out), *delayed recall* (recalling the ten-word list after a short delay), *orientation* (asking respondents the correct day of month, day of the week, month, and year), and *numeracy* (assessed by five arithmetical calculation tasks)[25-27]. A summary score of cognitive functioning was built by averaging the z-scores of the five items.

### Macroeconomic conditions

We use historical time-series data on annual Gross Domestic Product (GDP) per capita[28] as indicators of economic conditions. Data used for the analysis comprised the years 1959 to 2003. We separated the cyclical component from the secular trend in the log of GDP per capita for each country using the Hodrick-Prescott Filter (HP) with a smoothing parameter of 100.[29][30] We converted the cyclical component for each country into quartiles of deviation from the GDP trend. To derive information on country-specific booms and recessions over the study period, deviations from the trend in GDP falling in the highest quartile were classified as booms, while deviations falling in the lowest quartile was classified as recessions.[31] Appendix Figure 1 illustrates the exemplary sequence of booms and recessions in four countries. This information was linked to individual records from SHARE based on the year at every age since birth and country of birth, resulting in a variable indicating the number of recessions at every single age from year of birth up to age 49, the age for which comparable information on GDP was available for most individuals. Our analysis focuses on exposure to recessions at ages 25 to 49, with the earliest exposure of any respondent at age 25 in the year 1959. Exposure to economic fluctuation was summarized based on the number of years lived in booms and recessions at ages 25-34, 35-44, and 45-49.

Comparable data on GDP per capita for all countries was available only until 2003. For 21 % of participants aged 45 to 49 (*n* = 2,602), data on business cycle for the period after 2003 were therefore missing. We assigned a separate dummy for these respondents in order to incorporate them in the analysis, but excluding these individuals led to similar results.

### Individual level controls

All models include a set of linear splines for 5-year age-groups from age 50 to 74 to relax the assumption of linear aging-related decline in cognitive function. We included controls for being born before or after the Second World War in 1945 (WWII), country of residence, and measures of childhood conditions at age 10 to control for circumstances which may have influenced cognitive functioning independently of economic fluctuations, including: (a) self-rated health, (b) material deprivation based on items available at the parental home (e.g. a fixed bath, water supply or central heating), (c) self-reported diagnosis of major childhood illnesses, (d) occupation of main breadwinner, (e) the number of books at home and (f) self-rated mathematical and (g) language skills. We also controlled for educational attainment (primary or less, secondary or tertiary) based on the International Standard Classification of Education (ISCED) [32] and for respondents' first occupation, based on four major groups of the International Standard Classification of Occupations (ISCO-88).[33]

### Life-course occupational class mobility and working conditions

Data on employment histories came from the 2008/09 wave and covered the entire adult life starting from age of leaving full-time education (or age 15 for those without any schooling) until year of interview or exit from the labour market. Using the life-grid History Event Calendar, individuals were asked to report each paid job that lasted for 6 months or more. For each job, participants reported the year the job started; the occupation that best described the job based on the ISCO-88; whether job was part- or full-time; changes between part- and full-time during each job spell, and year and reason the job ended. We constructed a database indicating for every age between 25 and 49 whether an individual was working, and, across 10-year age categories, indicators of (a) downward occupational class mobility at least once; (b) multiple changes between full-time and part-time in a single job spell (as opposed to permanently working full- or part-time); (c) permanently working part-time in a given job; (d) job loss due to lay-off or plant/office being closed down at least once; (e) employment gaps due to reasons other than lay-off or plant/office being closed down. We then linked this information with data on the number of booms and recessions individuals experienced every decade of life.

### Statistical analysis

All analyses were stratified by gender. We analysed the association between cognitive functioning at ages 50 to 74 and number of recessions during the age intervals 25-34, 35-44 and 45-49 using linear regression. This approach exploits the fact that economic conditions at ages 25 to 49 are to a large extent random since individuals have no direct influence on them. To control for differences across countries that could bias estimates, we estimated a country-fixed effect model exploiting within-country variation across cohorts. The country-fixed effect thus controls for all unmeasured differences across countries such as institutional characteristics, economic development or levels of cognitive functioning. Estimates can be interpreted as the association of an additional recession at each given age interval on cognitive functioning, controlling for differences across countries. To explore possible mechanisms between economic conditions and later cognitive function, we also used logistic regressions to model the association of economic booms and recessions with occupational class mobility and working conditions at every decade of life, adjusting for all confounders.

We report unstandardised regression coefficients and 95 % confidence intervals (CI). All analyses were conducted using calibrated sampling weights to account for potential selectivity bias generated by unit nonresponse, sample attrition, and mortality.[34] All analyses were conducted in Stata/SE 12.1.

## Results

**Figure 1:**
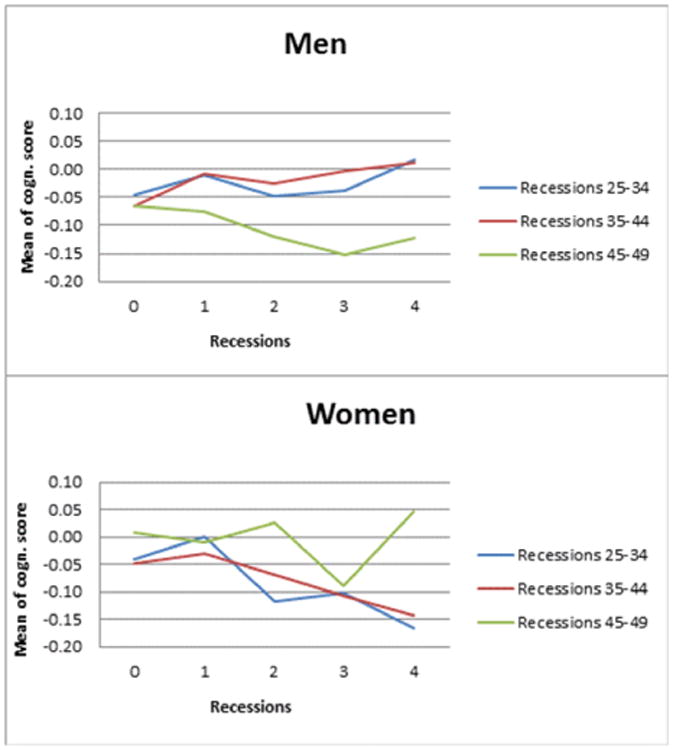
Predicted Means of Cognitive Function by Number of Recessions for Men and Women^a^ ^a^The two panels show the predicted z-score of cognitive functioning for men and women conditional on the number of recessions experienced at three different age intervals. Models include dummies for country of residence.

After excluding respondents with missing information, data of a total of 5,891 men and 6,129 women were included in the analyses. Number of recessions varied from 0.73 for men at ages 45-49 to 1.33 for women at ages 35-44 (Appendix Table 1). Number of booms was in no case related to later cognitive function. Figure 1 shows predicted means of cognitive function according to the number of recessions experienced at each decade of live, controlling for country dummies and age. Men experiencing no recession at ages 45-49 had a mean cognitive score of -0.07 at ages 50-74, compared to -0.12 for those who experienced 4 or more recessions at those ages. Similarly, women experiencing no recession at ages 25-34 had an average z-score of cognitive functioning of -0.05 at ages 50-74, whereas women having experienced four recessions in this age interval had an average z-score of -0.17.

Table 1 shows estimates of the association between recessions at different decades of life and z-scores of cognitive function, along estimates for confounders. Being born before WW II is associated with better cognitive scores for women (b = 0.12, p < 0.01), but not for men. Higher occupational status of first job is associated with lower cognitive function for both men and women. Higher education is associated with better cognitive function for both sexes. Worse self-rated skills in language and mathematics as a child are associated with lower cognitive function in both men and women.

Analyses included an extensive set of confounders and 5-year age splines. Among men, controlling for all confounders, each additional recession during ages 45-49 was associated with worse cognitive function at ages 50-74 (b = -0.06, CI -0.11, -0.01), while recessions at earlier ages were not associated with cognitive function. Among women, each additional recession at ages 25-34 or 35-44 was associated with worse cognitive function at ages 50-74 (b_25-34_ = -0.03, CI -0.04, -0.01; b_35-44_= -0.02, CI -0.04, -0.00). See Figure 1 for predicted means of cognitive function according to the number of recessions.

**Figure 2:**
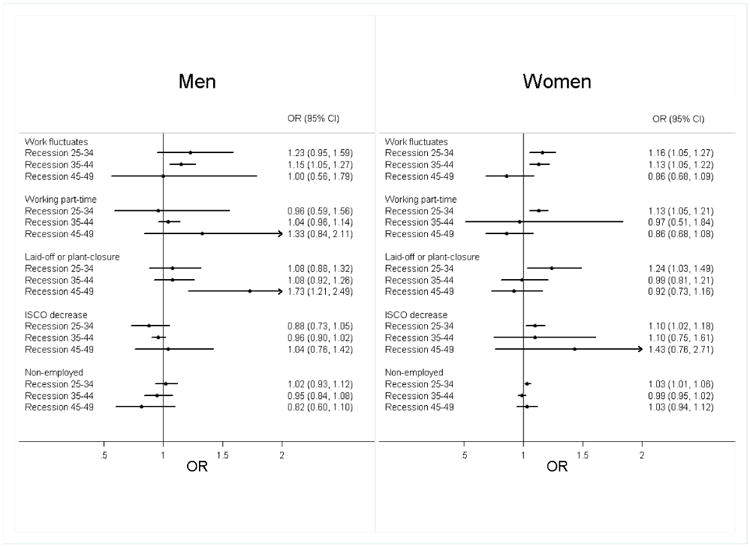
Associations of Recessions With Working Conditions (Occupational Class Mobility and Working Conditions)^a^ ^a^Abbreviations: OR, odds ratio; 95% CI, 95% confidence interval of double-sided test; P, p-value; ISCO, International Standard Classification of Occupations. The graphs show the odds ratios of one recession experienced at each age interval on experiencing a respective working condition in this age interval for men and women, after adjusting for all confounders. Odds ratios on the right side of the vertical line indicate increased likelihood to have experienced one of the five working conditions. All models were run separately for each type of working condition and age-interval and include the same individual-level covariates as in Table 1 as well as dummies for the country of residence (coefficients not shown).

Appendix Table 2 shows frequencies of selected working trajectories for men and women. Downward occupational mobility and job loss due to lay-off or plant closing is equally frequent in both men and women between ages 25 and 49 (for each decade less than 6%). Other changes in working conditions are more frequent for women than for men: multiple changes between full- and part-time work (men: < 3 % in each decade, women: 7.5 - 28.1 %), working part-time (men: < 3 %, women: 21.7 - 27.8 %), unemployment due to reasons other than lay-off or plant closing (men: 7.8 - 13.5 %, women: 38.2 - 54.3 %). . We first report on the association between each additional recession experienced at each age interval and unfavourable working condition at that same age interval after adjusting for all confounders. Among men, an additional recession experienced at ages 25-34 was not associated with working conditions, while a recession at ages 35-44 was associated with an increased odds of multiple changes between full-time and part-time working in that decade (OR = 1.15, 95 % CI 1.05, 1.27; Figure 2). A recession at ages 45-49 was associated with an increased odds of having lost a job due to layoff or plant/office closure (OR = 1.73, 95 % CI 1.21, 2.49). Among women, an additional recession at ages 25-34 was associated with worse labour market outcomes for all five indicators (Figure 2).

**Figure 3:**
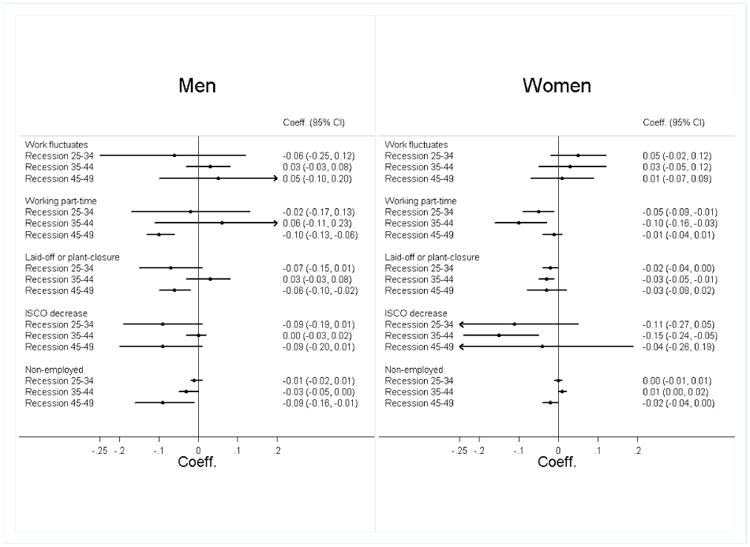
Associations of Working Conditions and Cognitive Function for Men and Women at Three Age Intervals ^a^ ^a^Abbreviations: Coeff., regression coefficient; 95% CI, 95% confidence interval of double-sided test; P, p-value; ISCO, International Standard Classification of Occupations. The graphs show the regression coefficients associated with experiencing a respective working condition in this age-interval and cognitive functioning after adjusting for all confounders. All models were run separately for each type of working condition and age-interval and include the same individual-level covariates as in Table 1 as well as dummies for the country of residence (coefficients not shown).

Next, we report on the associations of unfavourable working conditions with (lower) cognitive function. For men, job loss due to lay-off or plant/office closure, which was associated with number of economic recessions in that interval, was also associated with worse cognitive function at older age (b = -0.06, CI -0.01, -0.02; Figure 3). Among women, of the five indicators that showed an association of additional recessions at ages 25-34, two indicators – working part-time and job loss due to lay-off or plant closure – were in turn associated with significantly lower cognitive function at older age. At ages 35-44, an additional recession was also associated with more job instability as measured by higher odds of changing between full-time and part-time (OR = 1.13, 95 % CI 1.05, 1.22). Three of the labour market changes at ages 35-44 in women – working part-time, losing a job due to plant/office closure, and downward occupational mobility – were associated with worse late-life cognitive function.

## Discussion

Our study was motivated by previous evidence that working conditions are associated with later-life cognitive function and decline. Our findings provide evidence that economic recessions experienced at vulnerable periods in mid-life are associated with decreased later-life cognitive function, and that part of this association may operate through the link of recessions with working conditions and labour market trajectories. Men who experienced an additional economic recession at ages 45-49 fared worse cognitive outcomes later in life, which could potentially be due to higher likelihood of job loss due to lay-off or plant closure at these ages. Among women, experiencing an additional recession at ages 25-44 was also associated with poorer cognitive outcomes, which may be explained by their higher rates of job loss due to lay-off or plant/office closure, less stable job careers, and higher likelihood of downward occupational mobility associated with recessions.

### Explanation of results

Life course theory suggests that individuals may be more susceptible to environmental influences during certain development stages.[18] In this regard, our findings provide preliminary evidence on associations between macroeconomic shocks during working life and later-life cognitive function, extending earlier studies on associations between cognitive function at older age and economic conditions during childhood, [13, 14] and associations between later-life cognitive function and contextual level information, such as neighbourhood socioeconomic status,[35, 36] schooling laws,[37] and retirement policies.[38, 39] Although more evidence is needed to assess whether associations observed in our study are causal, our findings suggest that potentially unanticipated macroeconomic shocks during vulnerable periods in mid-life may affect an individual's potential to accumulate cognitive reserve. A possible explanation is that macro-economic conditions shape mid-life working conditions associated with late-life cognitive function.[2-10] However, our results suggest that men's susceptibility to macro-economic shocks may be larger at relatively late stages of the working career, while women are more susceptible to long-run effects on cognitive function if experiencing recessions during early- and mid-adulthood.

Findings for women suggest that early and mid-adulthood recessions are associated with unfavourable changes in working conditions. If causal, this may suggest that the association between economic recessions and cognitive function may operate to some extent through fluctuations in working time, lay-offs and plant closures. Previous evidence suggests that labour market participation among women is strongly influenced by the economic climate, with women during economic downturns being significantly more likely to be out of the labour market [40] or in non-standard employment,[41] which may reduce their life-time exposure to cognitively stimulating work-related activities. For younger workers, transitions into non-standard employment during economic downturns are more likely than for older workers.[41] This may account for the stronger association of early- and mid-life recessions with late-life cognitive function among women, while recessions experienced at ages 45 to 49 were not associated with cognitive function.

Our findings suggest that, among men, economic recessions in the later stages of mid-adulthood (ages 45-49) are critically linked to lower cognitive function after age 50, potentially due to the effect of recessions at these ages on the risk of job loss. Although young workers faced with a major recession may suffer long-lasting reductions in earnings and reduced labour market opportunities,[15] they are likely to return to work as economic conditions improve.[41] In contrast, for older workers job loss in the late stages of the working career may become an involuntary ‘pathway to retirement’, with older workers often leaving the labour market permanently during economic downturns [42] or after involuntary job loss [43] with reduced access to cognitively stimulating activities. This may explain why recessions in late-adulthood lead to larger reductions in cognitive function at older age, but not for early- and mid-adulthood recessions. An alternative pathway may lead from job loss to depressive symptoms and stress, which in turn are associated with reduced cognitive function in older ages.[44] The fact that economic recessions in later stages of adulthood were linked to cognitive function in men, but not in women, may stem from gender differences in occupational mobility. Previous research suggests that occupational mobility and associated wage gains are larger for men than for women.[45] Mid-life working careers of women have been and still are largely different from those of men, and women during middle age may be out of the labor market more often than men for different reasons. [46] Thus, a potential explanation is that recession effects on occupational mobility affect men more than they affect women. As a result, men who experience less favourable economic conditions in late mid-adulthood may accumulate less cognitive reserve as they approach older age.

The association between one additional recessions and cognitive functioning (females: b_25-34_ = -0.03; b_35-44_ = -0.02; males: b_45-49_ = -0.06) may seem relatively small compared to associations between cognitive function and other indicators, e.g. with higher education (females b_secondary education_=0.22; males b_secondary education_= 0.18). However, the association between one additional recession and cognitive function has a similar magnitude as one additional year of age with cognitive decline after age 60 (both sexes after age 60: b_age splines_ = -0.01 to -0.04).

### Limitations

The main strength of our study was the linkage between individual-level data on work histories, cognitive function and macro-economic shocks across European countries. However, several limitations should be considered. The first and most prominent limitation of our study is the fact that the abatement of selection, which we addressed by investigating the relationship between macro-economic conditions and cognitive function, is no proof for causality. However, our results provide tentative support for the hypothesis that economic recessions are associated with cognitive function possibly through changes in working conditions.

We investigated several mechanisms by which economic recessions may influence later-life cognitive function, however, more specific mechanisms such as reduced occupational complexity, social participation, or earnings could not be assessed. Strength of our study is the possibility to examine the role of national economic conditions on cognitive function on the basis of comparable data across countries. However, we were unable to appropriately assess potential differences across countries in magnitude and societal impact of recessions. Our findings are only applicable for the cohorts working during the post-WW II period in the European countries under investigation. Part of our sample was born before WW II and has experienced a dramatic and highly adverse historical period during childhood and/or adolescence. This cohort may have been severely affected in terms of nutrition, parental affluence, family and social networks, and quantity and quality of education before entering the work force. We addressed this by including a dummy for being born before, during or after the WW II, thus controlling for the effect of this shock on cognitive outcomes. Nevertheless, future studies are necessary to assess whether associations observed in our study might differ for cohorts differently affected by WW II.

### Conclusions

To our knowledge, this is the first study to show that economic recessions experienced at vulnerable ages in early and mid-adulthood are associated with lower cognitive function at older ages. Our findings also suggest that economic recessions during this period are associated with several labour market outcomes. If replicated, policies that encourage women to enter and remain attached to the labour market through early- and mid-adulthood, such as policies on schooling, maternity leave and childcare support, may have unanticipated positive effects on female cognitive function. Similarly, policies that enable men to return to work or remain engaged in productive activities at age 45 and beyond may be important in accumulating cognitive reserve. Preliminary evidence presented in this paper suggests that for older men, the later stages of their career may be more prone to economic recessions and, in turn, offer great potential to increase cognitive reserve, than earlier stages. More evidence is needed to assess whether specific policies that buffer the impact of economic downturns on labour market outcomes bring benefits to cognitive functioning in older age.

## Figures and Tables

**Table 1:** Linear Regressions of Number of Recessions at Ages 25-34, 35-44, and 45-49 on Cognitive Function at Ages 50-74, Controlling for First Occupation, Childhood Self-Rated Health, Number of Diseases, Number of Injuries, Number of Mental Conditions, School Performance, Parental Occupation, Number of Books in Parental Household at Age 10, and Education^a^

	Men			Women		
	Coeff.	95% CI	P	Coeff.	95% CI	P
Recessions						
Ages 25-34	0.01	(-0.02, 0.04)	0.54	-0.03	(-0.04, -0.01)	< 0.01
Ages 35-44	-0.01	(-0.06, 0.04)	0.71	-0.02	(-0.04, -0.00)	0.04
Ages 45-49	-0.06	(-0.11, -0.01)	0.02	-0.02	(-0.05, 0.01)	0.11
Age (Splines)						
50-54	0.19	(-0.02, 0.39)	0.07	-0.1	(-0.22, 0.02)	0.10
55-59	0.04	(-0.04, 0.12)	0.30	-0.03	(-0.11, 0.05)	0.43
60-64	-0.04	(-0.07, -0.00)	0.03	-0.01	(-0.04, 0.02)	0.45
65-69	-0.01	(-0.04, 0.02)	0.49	-0.04	(-0.05, -0.03)	< 0.01
70-74	-0.02	(-0.04, 0.00)	0.11	-0.02	(-0.03, 0.00)	0.08
born before World War II (1945)	0.04	(-0.10, 0.17)	0.55	0.12	(0.06, 0.17)	< 0.01
Early-life socio-economic characteristics 1st job International Standard Classification of Occupations (ISCO) (ref.: low skilled blue collar)										
High skilled blue collar	0.03	(-0.01, 0.07)	0.18	-0.05	(-0.13, 0.03)	0.2
Low skilled white collar	-0.11	(-0.17, -0.05)	< 0.01	-0.17	(-0.26, -0.09)	< 0.01
High skilled white collar	-0.13	(-0.17, -0.08)	< 0.01	-0.16	(-0.22, -0.09)	< 0.01
Educational level (ref.: primary or less)						
Secondary education	0.18	(0.09, 0.26)	< 0.01	0.22	(0.17, 0.28)	< 0.01
Post-secondary education	0.32	(0.26, 0.38)	< 0.01	0.31	(0.24, 0.38)	< 0.01
Childhood health						
(Very-) bad self-rated health as child (ref.: fair or (very-) good	0.06	(-0.00, 0.12)	0.06	-0.04	(-0.12, 0.04)	0.33
1+ infectious disease during childhood	0.05	(-0.01, 0.11)	0.09	0.03	(-0.03, 0.10)	0.29
1+ physical injury during childhood	-0.12	(-0.19, -0.04)	0.01	0.01	(-0.01, 0.03)	0.15
Mental condition during childhood	-0.10	(-0.38, 0.18)	0.44	0.06	(-0.05, 0.16)	0.25
No. of books at home (ref.: none or very few (0-10)						
Enough to fill one shelf (11-25)	0.10	(0.04, 0.17)	0.01	0.02	(-0.03, 0.08)	0.37
Enough to fill one bookcase (26-100)	0.09	(0.00, 0.19)	0.04	0.10	(0.04, 0.15)	< 0.01
Enough to fill two bookcases (101-200)	0.24	(0.12, 0.35)	< 0.01	0.11	(-0.02, 0.23)	0.08
Enough to fill two or more bookcases (more than 200)	0.24	(0.15, 0.32)	< 0.01	0.12	(0.00, 0.23)	0.05
Main occupation of the breadwinner during childhood (ref: low skilled blue collar)										
High skilled blue collar	-0.02	(-0.10, 0.06)	0.52	0.00	(-0.05, 0.05)	0.99
Low skilled white collar	-0.04	(-0.14, 0.07)	0.45	-0.02	(-0.10, 0.07)	0.70
High skilled white collar	0.03	(-0.11, 0.17)	0.63	0.03	(-0.05, 0.12)	0.38
Childhood math-skills compared to peers (ref.: much better)						
Better	-0.05	(-0.10, -0.01)	0.02	-0.01	(-0.10, 0.08)	0.84
About the same	-0.16	(-0.21, -0.11)	< 0.01	-0.14	(-0.24, -0.05)	0.01
Worse	-0.34	(-0.48, -0.21)	< 0.01	-0.25	(-0.40, -0.09)	0.01
Much worse	-0.24	(-0.38, -0.10)	< 0.01	-0.37	(-0.50, -0.24)	< 0.01
Childhood language-skills compared to peers (ref.: much better)						
Better	-0.02	(-0.09, 0.05)	0.48	-0.05	(-0.11, -0.00)	0.04
About the same	-0.07	(-0.13, -0.00)	0.04	-0.10	(-0.16, -0.04)	< 0.01
Worse	-0.09	(-0.19, 0.00)	0.05	-0.16	(-0.23, -0.09)	< 0.01
Much worse	-0.32	(-0.44, -0.20)	< 0.01	-0.33	(-0.44, -0.21)	< 0.01

Abbreviations: Coeff., regression coefficient; 95% CI, 95% confidence interval of double-sided test; P, p-value; WWII, World War II; ISCO, International Standard Classification of Occupations; SRH – self-rated health.

a Notes: N = 12,020. All models include dummies for country of residence (coefficients not shown).

**Figure:**
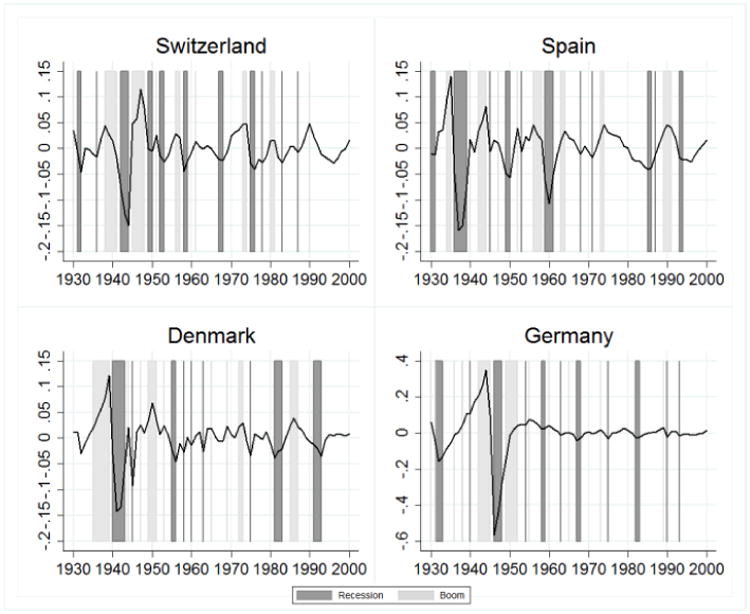
Booms and Recessions in Four European Countries (1930-2003)^a^ ^a^The Figure shows annual deviations from the smoothed trend of (log) GDP per capita for four representative countries (Germany, Denmark, Spain and Switzerland) for the years 1930 to 2003 as well as the years in which there was a recession or a boom, defined as deviations from the trend falling in the lowest or highest country-specific quartile respectively. A positive deviation indicates a cyclical upturn and a negative deviation a cyclical downturn. In addition to the annual deviations from the smoothed trend, the light-grey shaded areas show if there was a boom and the dark-grey shaded bars show if there was a recession in a specific year. As the deviations from the smoothed trend highlight, in all four countries there are marked fluctuations in the development of (log) GDP per capita over this time-period, thus making it possible to identify booms and recessions from the data.

## What is already known on this subject?

- Labour market engagement and stimulating working environments increase cognitive reserve and predict better cognitive function at older age.
- Fluctuations in the national economy shape labour market opportunities and outcomes, but the link between economic fluctuations during working ages and cognitive function in later ages has not yet been established.

## What this study adds

- Economic recessions experienced at vulnerable ages in mid-adulthood are associated with lower cognitive function at older ages.
- Recessions during mid-life are associated with cognitive function in older age, possibly via their impact on the likelihood of job loss for both men and women, and the lower likelihood of labour market engagement and downward occupational mobility among women.
- If findings are replicated, policies that encourage women to enter and remain attached to the labour market through early- and mid-adulthood, and policies that enable men to return to work or remain engaged in productive activities during the late stages of their career may bring benefits to cognitive functioning in older age.

## Appendix Table 1

**Table:** Sample Characteristics for Men and Women (N = 12,020)

	Men (N = 5,891)		Women (N = 6,129)	
	N (mean)	% (SD)	N (mean)	% (SD)
Cognition Z-Score	(0.00)	(0.63)	(0.00)	(0.65)
Number of Recessions				
At Ages 25-34	(1.31)	(1.27)	(1.32)	(1.28)
At Ages 35-44	(1.29)	(1.15)	(1.33)	(1.17)
At Ages 45-49	(0.73)	(1.31)	(0.77)	(1.36)
Age	(63.24)	(6.04)	(62.68)	(5.95)
born before World War II (1945)	2,822	47.90	2,711	44.23
1st job International Standard Classification of Occupations (ISCO)				
Low skill blue	1,312	22.27	966	15.76
High skilled blue collar	1,100	18.67	2,884	47.05
Low skilled white collar	1,868	31.71	617	10.07
High skilled white collar	1,611	27.35	1,662	27.12
Educational level				
Primary or less	2,170	36.84	2,600	42.42
Secondary education	1,982	33.64	1,908	31.13
Post-secondary education	1,739	29.52	1,621	26.45
Childhood health				
(Very-) bad self-rated health as child (ref.: fair or (very-) good	431	7.32	544	8.88
1+ infectious disease during childhood	4,925	83.60	5,441	88.77
1+ physical injury during childhood	1,584	26.89	1,771	28.90
Mental condition during childhood	73	1.24	115	1.88
No. of books at home during childhood				
None or very few (0-10)	2,449	41.57	2,165	35.33
Enough to fill one shelf (11-25)	1,311	22.24	1,467	23.93
Enough to fill one bookcase (26-100)	1,305	22.16	1,538	25.09
Enough to fill two bookcases (101-200)	404	6.86	479	7.81
Enough to fill two or more bookcases (more than 200)	422	7.16	481	7.84
Main occupation of the breadwinner during childhood				
Low skilled blue collar	1,562	26.50	1,680	27.41
High skilled blue collar	2,701	45.87	2,617	42.70
Low skilled white collar	845	14.34	901	14.70
High skilled white collar	784	13.30	931	15.18
Self-reported childhood math skills compared to peers				
Much better	782	13.28	591	9.65
Better	1,661	28.20	1,412	23.03
About the same	2,733	46.40	3,217	52.49
Worse	595	10.12	748	12.20
Much worse	119	2.01	161	2.63
Self-reported childhood language skills compared to peers				
Much better	536	9.10	794	12.96
Better	1,384	23.50	1,875	30.59
About the same	2,928	49.71	2,858	46.64
Worse	913	15.49	525	8.56
Much worse	129	2.19	77	1.26

## Appendix Table 2

**Table:** Life-Time Occupational Class Mobility and Working Conditions at Ages 25-34, 35-44, and 45-49 Derived from Retrospective Employment Histories Collected in 2008/9

	Men (N = 5,195)		Women (N = 5,557)	
	N (mean)	% (SD)	N (mean)	% (SD)
Downward occupational class mobility at least once				
25-34	293	5.64	250	4.50
35-44	174	3.34	194	3.49
45-49	100	1.92	108	1.95
Changed multiple times between full-time and part-time in a single job				
25-34	79	1.53	417	7.51
35-44	90	1.74	1,563	28.13
45-49	117	2.25	1,501	27.01
Always worked part-time				
25-34	107	2.06	1,206	21.71
35-44	99	1.90	1,547	27.83
45-49	111	2.14	1,500	26.99
Job loss due to lay-off or plant/office being closed down at least once				
25-34	73	1.40	235	4.23
35-44	119	2.30	282	5.08
45-49	145	2.79	271	4.88
Employment gap due to reasons other than lay-off or plant/office being closed down				
25-34	699	13.45	3,017	54.30
35-44	406	7.81	2,625	47.23
45-49	428	8.23	2,124	38.23

## List of Abbreviations

SHARE: Survey of Health, Ageing and Retirement in Europe
OR: odds ratio
CI: confidence interval
HP: Hodrick-Prescott Filter
ISCED: International Standard Classification of Education
ISCO: International Standard Classification of Occupations
WWII: World War II
